# Insights into epigenetic landscape of recombination-free regions

**DOI:** 10.1007/s00412-016-0574-9

**Published:** 2016-01-22

**Authors:** Pasquale Termolino, Gaetana Cremona, Maria Federica Consiglio, Clara Conicella

**Affiliations:** CNR, National Research Council of Italy, Institute of Biosciences and Bioresources, Research Division Portici, Via Università 133, 80055 Portici, Italy

**Keywords:** DNA methylation, Histone acetylation, Histone methylation, Heterochromatin, Euchromatin, Meiosis

## Abstract

Genome architecture is shaped by gene-rich and repeat-rich regions also known as euchromatin and heterochromatin, respectively. Under normal conditions, the repeat-containing regions undergo little or no meiotic crossover (CO) recombination. COs within repeats are risky for the genome integrity. Indeed, they can promote non-allelic homologous recombination (NAHR) resulting in deleterious genomic rearrangements associated with diseases in humans. The assembly of heterochromatin is driven by the combinatorial action of many factors including histones, their modifications, and DNA methylation. In this review, we discuss current knowledge dealing with the epigenetic signatures of the major repeat regions where COs are suppressed. Then we describe mutants for epiregulators of heterochromatin in different organisms to find out how chromatin structure influences the CO rate and distribution.

## Introduction

**Fig. 1:**
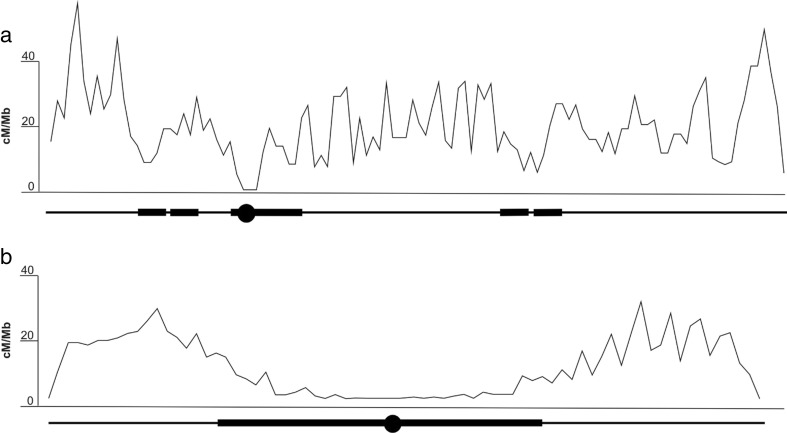
Recombination rate variation along human (**a**) and tomato (**b**) chromosome 12. Below each graph, chromosome structure is represented by euchromatin (*thin line*) and heterochromatin (*thick line*). Data have been extrapolated from Myers et al. (2005) (**a**) and The Tomato Genome Consortium (2012) (**b**)

Genome architecture is shaped by gene-rich and repeat-rich regions also known as euchromatin and heterochromatin, respectively. Repeat sequences exist at a high number in the genomes of eukaryotes and are localized mostly in centromeres, telomeres, transposable elements, ribosomal (rRNA), and transfer RNA (tRNA) loci. Under normal conditions, the repeat-containing regions undergo little or no meiotic crossover recombination (Fig. 1) (Chen et al. 2008; Pan et al. 2011).

Meiotic recombination is initiated by the SPO11 transesterase-dependent DNA double-strand breaks (DSBs) that are repaired as crossovers (COs), i.e., by reciprocal exchanges between the homologous chromosomes. In many organisms, more DSBs are formed than COs, thereby indicating alternative pathways of DSB repair including non-crossover (NCO), i.e., non-reciprocal exchange and inter-sister repair (IS) (Mercier et al. 2015; de Massy 2013). Multiple layers of CO control exist in most organisms. Firstly, recombination events are non-randomly distributed along chromosomes and they cluster in small regions called hot spots. Secondly, the phenomenon of CO interference leads nearby COs, so they occur further apart on a chromosome than would be expected by chance (Berchowitz and Copenhaver 2010). Finally, CO homeostasis maintains nearly constant CO number per meiosis despite variation in DSB numbers (Martini et al. 2006).

The mechanisms that are responsible of CO suppression in repeat regions can operate at different levels limiting the chromatin accessibility (Ben-Aroya et al. 2004), preventing DSBs, and repairing DSBs through NCO or IS. In budding yeast, it has been shown that open chromatin structure is required for SPO11 to access DNA for generating meiotic DSBs (Berchowitz et al. 2009; Pan et al. 2011). Vice versa, heterochromatic regions that have a closed conformation constrain SPO11 access (Pan et al. 2011). Notwithstanding the suppression, DSBs likely occur in repeats. In case that DSBs are repaired as COs within repeats, the genome integrity is at risk. Indeed, COs between repeats can promote non-allelic homologous recombination (NAHR) resulting in deleterious genomic rearrangements associated with human diseases (Shaw et al. 2002; Sasaki et al. 2010).

The assembly of heterochromatin is driven by the combinatorial action of many factors including histones, their modifications, and DNA methylation. Here, we discuss current knowledge concerning the epigenetic signatures of the major repeat regions where COs are suppressed. Then, we describe mutants for epiregulators of heterochromatin in different organisms to find out how chromatin structure influences the CO rate and distribution.

## Where COs are suppressed

### Centromeres

Centromeres are the chromosomal parts to which spindle microtubules become attached ensuring proper segregation during mitosis and meiosis. Centromeres are universally marked by the histone H3 variant CENH3, also known as CENP-A in mammals, which replaces canonical histone H3 in centromeric nucleosomes. In most eukaryotes, CENH3-containing chromatin is flanked by pericentromeric heterochromatin (Stimpson and Sullivan 2010). The pericentromeric domains are generally enriched in DNA methylation and histone modifications such as H3K9me2 (Simon et al. 2015). Except for budding yeast, centromeres are not characterized by specific DNA sequences. In budding yeast, the centromeric region is short (≈125 bp) and harbors a single nucleosome (Furuyama and Biggins 2007), whereas in most animals and plants, centromeric regions consist of several megabases (e.g., 1–2.5 Mb in human) (Steiner and Henikoff 2015). In the worm *Caenorhabditis elegans*, the holocentromere is formed by point centromeres distributed along the length of the chromosomes (Steiner and Henikoff 2014).

Unlike organisms with a localized centromere, during meiosis of *C. elegans*, the position of the centromere is dictated by the site of the CO, which is normally a single one per homolog pair (Schvarzstein et al. 2010). This exceptional behavior is likely due to the organization of holocentric chromosomes. Overall, the centromeric regions have a low recombination rate at several times below the genome average. In budding yeast, the nucleotide resolution map evidenced that DSBs are suppressed within 5-kb region around centromeres (Pan et al. 2011). Similarly, COs are almost depleted at centromeres in *Arabidopsis*, maize, and rice (Copenhaver et al. 1999; Drouaud et al. 2006; Shi et al. 2010; Si et al. 2015). However, NCOs were found in maize centromeric regions at a similar rate to the chromosome arms, thereby suggesting that DSBs are not prevented. In human, a drop of recombination rate estimated by CO maps was observed close to the centromeres (Myers et al. 2005; Lu et al. 2012; Hou et al. 2013). The mechanism by which centromeric recombination could interfere with chromosome function is not established. Presumably, COs too near the centromere are a constraint for kinetochore assembly (Ellermeier et al. 2010) with negative effects on chromosome segregation (Talbert and Henikoff 2010). For instance, centromeric COs can result in meiotic missegregation in fission yeast (Hall et al. 2003) and in humans where they can cause severe birth defects, such as trisomy 21 (Lamb et al. 1996; Ottolini et al. 2015).

### Telomeres

Telomeres protect the ends of chromosomes. In a wide range of organisms, telomeres consist of short tandem repeats. In budding yeast, telomeres are short (∼300 bp of single repeats) and nucleosome-free while sub-telomeric repetitive elements, called TAS (telomere associated sequences), are likely organized in nucleosomes (Wellinger and Zakian 2012). On the other hand, telomeres of higher eukaryotes have extremely variable length (i.e., ∼2–15 kb of hexameric repeats in human somatic cells) and are organized in tightly packaged nucleosomes (Pisano et al. 2008). A conserved feature of telomeres is their enrichment in heterochromatic marks. For example, the mammalian telomeres are enriched in H3K9me3, H4K20me3, and hypoacetylated H3 and H4 histones. The function and structural integrity of telomeres depends on their repressive chromatin structure (reviewed in Peuscher and Jacobs 2012; Galati et al. 2013). Indeed, a decrease of above-mentioned heterochromatic marks in mice mutants result in aberrantly increased telomere length and chromosomal instability (García-Cao et al. 2004; Gonzalo et al. 2005; Palacios et al. 2010). Telomeres are suppressed for meiotic recombination. In budding yeast, 20-kb region adjacent to telomeres exhibit a significant lower meiotic DSB rate (Pan et al. 2011) consistent with previous observations about the reduction of COs (Barton et al. 2008; Chen et al. 2008). Interestingly, sub-telomeric regions show high levels of COs in the male meiosis of plants and mammals (Saintenac et al. 2009; Giraut et al. 2011; Paigen et al. 2008; de Boer et al. 2015). In the latter, sub-telomeric COs are promoted on both autosomes and sex chromosomes of the males. In particular, COs need to be formed in the sub-telomeric pseudoautosomal region of PAR, that is a very small region of homology between the X and Y chromosomes, to ensure their correct segregation (Kauppi et al. 2011; Hinch et al. 2014). In hermaphroditic plants lacking sex chromosomes, the sub-telomeric localization of recombination events in male meiosis could be associated with a selective advantage of the male gametophytes (Lenormand and Dutheil 2005). Conversely, the regions close to telomeres have a lower recombination in female meiosis than in male (Giraut et al. 2011; Paigen et al. 2008; de Boer et al. 2015). It is a widespread phenomenon termed “heterochiasmy” that is the difference in CO number and position between male and female meiosis (Lenormand 2003). In mice, the sex-specific differences in COs principally reflect differences in the recombination outcome, COs vs NCOs, between males and females (de Boer et al. 2015).

Telomeric COs may be detrimental to chromosome segregation (Ross et al. 1996; Su et al. 2000). In humans, the occurrence of a telomeric CO is associated with non-disjunction of chromosome 21 in female meiosis leading to Down syndrome (Oliver et al. 2012). The current model suggests that COs near telomeres have a negative effect on the cohesion between CO and the chromosome end, thereby threatening the bivalent maintenance in oocytes over the prolonged prophase I.

### Ribosomal DNA

Ribosomal DNA (rDNA) has the essential role of encoding most of cellular RNA (Moss et al. 2007). It is composed of tandemly repeated genes and non-coding intergenic spacers (IGS), clustered in regions which are frequently sub-telomeric (McStay and Grummt 2008). In budding yeast, the rDNA cluster is organized into a single array of 100–200 units, whereas rDNA units can be repeated up to thousands of times in other organisms (Eickbush and Eickbush 2007). An rDNA array contains both transcriptionally active and transcriptionally silent repeats. For instance, ∼10 % of rDNA copies are usually active in *Arabidopsis* (Dvořáčková et al. 2015). The chromatin marks detected in a rDNA region are those typically associated to euchromatin or to heterochromatin depending on the activity of individual units (Bierhoff et al. 2014). In budding yeast, rDNA gene clusters are repressed for meiotic recombination (Gottlieb and Esposito 1989). Consistently, meiotic DSBs appeared almost absent from rDNA (Blitzblau et al. 2007) occurring 75-fold below genome average (Pan et al. 2011). Thus, the suppression of meiotic recombination at rDNA loci can mostly be attributed to the suppression of meiotic DSB formation. Additionally, some findings suggest that when DSBs are formed at rDNA loci they are repaired by IS, which also works to suppress NAHR (Petes 1980; Li et al. 2014).

## Chromatin landscape of recombination suppression: histone variants and post-translational modifications

Histones and their modifications have a fundamental role in the assembly of chromatin domains (Kouzarides 2007). In the context of gene-rich regions, the prominent chromatin mark of meiotic recombination initiation is the histone H3 lysine 4 trimethylation (H3K4me3) in yeast (Borde et al. 2009; Sommermeyer et al. 2013; Acquaviva et al. 2013a) and mouse (Buard et al. 2009; Brick et al. 2012). Comprehensive literature reviewed the relationship between histone H3K4me3 and DSB formation (Borde and de Massy 2013; Acquaviva et al. 2013b). In *Arabidopsis*, in addition to H3K4me3, histone variant H2A.Z is found to be enriched at CO sites. In a mutant, which fails to deposit H2A.Z in the nucleosome, CO frequency is reduced (Choi et al. 2013).

Histone marks of repeat-associated chromatin differ between organisms. However, enrichment of histone H3 lysine 9 methylation (H3K9me) and wide histone de-acetylation are common features of heterochromatin (Table 1) (Nakayama et al. 2001; Suka et al. 2001). In budding yeast, the silent information regulator (*Sir*) genes are required for assembly of heterochromatin. In particular, Sir2 deacetylates H4K16 near the telomeres and within the rDNA cluster (Robyr et al. 2002). Loss of *Sir2* alters the pattern of meiotic recombination (Gottlieb and Esposito 1989; Mieczkowski et al. 2007). Indeed, DSBs increased within 10 kb closest to telomeres and within the rDNA cluster, thereby avoiding the typical suppression. It was suggested that increased levels of acetylation in *sir2* mutant open the chromatin structure by allowing recombination machinery easier access to DNA. Consistently, specific hyperacetylation at H4K16 has been demonstrated in vitro to directly influence higher-order chromatin structure by inhibiting the formation of compact 30-nm chromatin fiber (Shogren-Knaak et al. 2006). On the other hand, an increased suppression of DSBs occur in 20–120 kb near-telomeric regions in *sir2* possibly determined by compensatory effects associated to interference. Although Sir2 prevents meiotic DSBs within rDNA, it has a DSB promoting effect on the rDNA borders, at the heterochromatin/euchromatin junctions. Since even DSBs adjacent to repetitive DNA can trigger NAHR, a border-specific protection system exists in budding yeast that counteracts DSB activities at rDNA borders. The buffer zones at the edges of the rDNA array are established by two interacting ATPases, Pch2 and Orc1, that prevent rDNA-proximal DSBs (Vader et al. 2011).

**Table 1:** Main features associated to the different chromatin domains in higher eukaryotes

Feature	Euchromatin	Heterochromatin
Structure	Loosely packed, open, accessible	Densely packed, closed, inaccessible
Composition	Mainly genes	Mainly repetitive elements
Activity	Expressed, active	Repressed, silent
DNA methylation	Hypomethylation	Hypermethylation
Histone post-translational modifications	Hyperacetylation of H3 and H4, H3K4me1, H3K4me2, H3K4me3, H3K27me3	Hypoacetylation of H3 and H4 H3K9me2
Histone variants	H2AX, H2A.Z	H2A.Z, CENH3 (CENP-A)

H3K9me is enriched in domains of heterochromatin to maintain its silent and compact conformation (Lachner and Jenuwein, 2002; Mozzetta et al. 2015). A substantial amount of evidence suggests that H3K9me2 exerts a repressive role on meiotic recombination. In fission yeast, deletion mutants for components of Clr4–Rik1complex that methylates H3K9 in and near the centromeres had abundant centromeric recombination (Ellermeier et al. 2010). Acquisition of H3K9me2 at a targeted hotspot due to the increase in DNA methylation (see next section) is associated with recombination suppression in *Arabidopsis* (Yelina et al. 2015). The post-translational modifications of H3 at the same residue, lysine 9, i.e., methylation (H3K9me) or acetylation (H3K9ac), are antagonistic and have opposite effects on meiotic recombination. H3K9ac is found to mark regions that are proficient in meiotic recombination in fission yeast, *Arabidopsis*, and mice (Yamada et al. 2013; Perrella et al. 2010; Buard et al. 2009). Vice versa, in meiotic cells of mice, H3K9me3 was shown to be enriched in the recombinationally inactive allele relative to the active one at hotspot *Psmb9* (Buard et al. 2009). In *C. elegans*, proper accumulation of H3K9me2 on meiotic prophase chromosomes is driven by *HIM*-*17* which is essential for SPO11-induced DSBs (Reddy and Villeneuve 2004). However, timing of accumulation of cytologically detectable H3K9me2 does not correlate with DSB formation but instead with DSB repair. This evidence is consistent with an experiment in human mitotic cells showing a transient enrichment of H3K9me at DSBs. The authors suggested H3K9me to be critical for remodeling the damaged chromatin to allow efficient DNA repair (Ayrapetov et al. 2014).

## Chromatin landscape of recombination suppression: DNA methylation

DNA methylation is a widespread epigenetic mark of repeat sequences associated with heterochromatin in eukaryote genomes of fungi, plants, and animals. DNA methylation is also found within bodies of active genes as a conserved feature between plants and animals (Zemach et al. 2010). Recently, DNA methylation was discovered in model species such as fruit fly (Takayama et al. 2014) and roundworm (Greer et al. 2015).

In different organisms, DNA methylation plays a repressive role on meiotic recombination. In the fungus *Ascobolus immersus*, the controlled addition of cytosine methylation (5mC) in a recombination interval encompassing a hotspot evidenced that the increase of DNA methylation has an inhibitory effect on COs by over a 100-fold (Maloisel and Rossignol 1998). In *Arabidopsis* and maize, genome-wide analysis of hotspots show low levels of DNA methylation (Choi et al. 2013; Rodgers-Melnick et al. 2015). In particular, the *Arabidopsis* region including hotspots *3a* and *3b* is characterized by a very low value of 5mC in all sequence contexts (CG, CHG, CHH where H = A, T, or C) compared with the genome average. De novo DNA methylation directed to this region by siRNAs causes significant suppression of recombination within the hotspots (Yelina et al. 2015). Mutants of *Arabidopsis* for *METHYLTRANSFERASE1* (*MET1*), involved in the maintenance of 5mC methylation in CG context, and for *DECREASED DNA METHYLATION 1* (*DDM1*), that encodes a SWI2/SNF2-like chromatin-remodeling protein necessary for DNA methylation, highlight that loss of DNA methylation increases meiotic recombination in the gene-rich chromosome arms (Mirouze et al. 2012; Melamed-Bessudo and Levy 2012). The mutants, however, do not show any increase of COs in heterochromatin, mainly pericentromeric in *Arabidopsis* (Colomé-Tatché et al. 2012; Mirouze et al. 2012; Melamed-Bessudo and Levy 2012; Yelina et al. 2012). DNA methylation is not, evidently, the only player in the suppression of COs in the plant repeat-containing regions. The occurrence of an epigenetic “double-lock” system was suggested by Melamed-Bessudo and Levy (2012). They hypothesize that, besides methylation-related condensation, recombination is inhibited by an additional factor controlling chromatin structure in heterochromatic regions.

A recent study revealed a histone-guided mechanism for the establishment of DNA methylation (Guo et al. 2015). In *ddm1* heterochromatin, DNA methylation loss is connected to the decrease of H3K9me2 (Gendrel et al. 2002). Silenced hot spots, while increasing DNA methylation, gained H3K9me2 and nucleosome occupancy (Yelina et al. 2015). In *Arabidopsis*, the combination of changes for chromatin modifications including DNA methylation loss remodels the distribution of COs without affecting the total COs since the homeostatic effect is maintained. Particularly in *met1*, CO remodeling is suggested to be driven by loss of interfering COs from pericentromeric regions and increase in the euchromatic regions (Yelina et al. 2015).

In contrast with above-reported evidence, a positive correlation was found between germ-line DNA methylation and regional recombination rate in the human genome. In particular, DNA methylation increased in regions within recombination hot spots in male germ cells (Sigurdsson et al. 2009). This observation could be caused by the indirect effect of an increase in guanine and cytosine (GC) content at meiotic recombination hot spots due to the mechanism known as GC-biased gene conversion (Duret and Galtier 2009; Arbeithuber et al. 2015). Since CpG islands are the major sites of DNA methylation in mammals, this would result in higher methylation at recombination hot spots.

## Conclusions

**Fig. 2:**
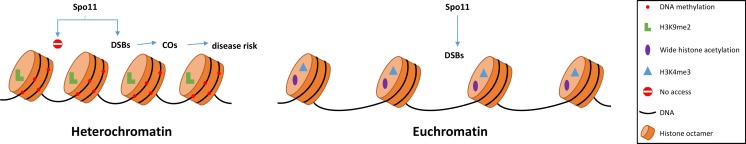
Epigenetic landscape view of repeat-rich (heterochromatin) and gene-rich regions (euchromatin) associated with meiotic recombination suppression and proficiency, respectively. SPO11, an evolutionarily conserved protein, is the enzyme that promotes DNA DSBs and initiation of meiotic recombination

Direct and indirect evidence collected in the different organisms thus far highlight that epigenetic landscape of repeats is related to the suppression of DSBs/COs. Heterochromatin-enriched epigenetic marks likely operate at different levels to avoid meiotic recombination. In detail, they can influence the higher-order heterochromatin structure to make it more close than euchromatin creating a physical barrier to recombination machinery (Fig. 2). Furthermore, epigenetic marks can act as heterochromatin signatures to filter the access to the protein complexes of recombination machinery differentially. Finally, they exclude the epigenetic marks that are enriched in DSB/CO prone regions. The high-resolution mapping of DSB/CO formation in mutants for epiregulators of heterochromatin in various species will clarify the epigenetic framework of the meiotic recombination suppression. However, generation of high-resolution maps of DSBs/COs, their integration with epigenomic maps, and with chromosome conformation in meiotic cells are challenges in multicellular organisms. Maps of meiotic DSBs are not available in most species due to technical constraints. In addition, recombination maps generally do not include repeat-rich regions. To date, maps of DSBs at nucleotide resolution are available in budding and fission yeasts (Pan et al. 2011; Fowler et al. 2014). In mice and human genomes, repeat regions have been excluded in the high-resolution maps of male meiotic DSBs (Brick et al. 2012; Pratto et al. 2014).
